# Oxidative stress

**DOI:** 10.1016/j.bpobgyn.2010.10.016

**Published:** 2011-06

**Authors:** Graham J. Burton, Eric Jauniaux

**Affiliations:** a Centre for Trophoblast Research, University of Cambridge, Cambridge, UK; b Academic Department of Obstetrics and Gynaecology, EGA Institute of Women Health, Royal Free and University College London Medical School, London, UK

**Keywords:** placenta, oxidative stress, antioxidants, miscarriage, pre-eclampsia

## Abstract

Considerable evidence implicates oxidative stress in the pathophysiology of many complications of human pregnancy, and this topic has now become a major focus of both clinical and basic science research. Oxidative stress arises when the production of reactive oxygen species overwhelms the intrinsic anti-oxidant defences. Reactive oxygen species play important roles as second messengers in many intracellular signalling cascades aimed at maintaining the cell in homeostasis with its immediate environment. At higher levels, they can cause indiscriminate damage to biological molecules, leading to loss of function and even cell death. In this chapter, we will review how reactive oxygen species are generated and detoxified in the human placenta, and what roles they may play at homeostatic concentrations. We will then consider their involvement in normal placental development, and in complications ranging from miscarriage to pre-eclampsia and premature rupture of the membranes.

## 1 Introduction

Oxygen is often referred to as the Janus gas, as it has both positive benefits and potentially damaging side-effects for biological systems. Reactivity allows oxygen to participate in high-energy electron transfers, and hence support the generation of large amounts of adenosine-5-triphosphate (ATP) through oxidative phosphorylation. This is necessary to permit the evolution of complex multicellular organisms, but also renders it liable to attack any biological molecule, be it a protein, lipid or DNA. Consequently, our body is under constant oxidative attack from reactive oxygen species (ROS). A complex system of antioxidant defences has evolved that generally holds this attack in balance. On occasions, however, this balance can be perturbed, leading to oxidative stress. Because of the multiple and diverse effects that oxygen toxicity can have on a cell, oxidative stress is best defined in broad terms as an alteration in the pro-oxidant–antioxidant balance in favour of the former that leads to potential damage.^1^ Oxidative stress is now recognised to play a central role in the pathophysiology of many different disorders, including complications of pregnancy.

The concept of a pro-oxidant–antioxidant balance is central to an understanding of oxidative stress for several reasons. Firstly, it emphasises that the disturbance may be caused through changes on either side of the equilibrium (e.g. abnormally high generation of ROS or deficiencies in the antioxidant defences). Secondly, it highlights the homeostatic concentrations of ROS. Although ROS first came to the attention of biologists as potentially harmful by-products of aerobic metabolism, it is now recognised that they play important roles as secondary messengers in many intracellular signalling pathways.^2^ Finally, the concept of a balance draws attention to the fact that there will be a graded response to oxidative stress. Hence, minor disturbances in the balance are likely to lead to homeostatic adaptations in response to changes in the immediate environment, whereas more major perturbations may lead to irreparable damage and cell death. The boundary between normal physiological changes and pathological insults is therefore inevitably indistinct.

The definition of oxidative stress provided above is necessarily broad because the outcome depends in part on the cellular compartment in which the ROS are generated. There are many potential sources of ROS, and the relative contributions of these will depend on the environmental circumstances prevailing. As the reactions of ROS are often diffusion-limited, the effects on cell function depend to a large extent on the biomolecules in the immediate vicinity. Different insults will therefore generate different outcomes.

A further feature of oxidative stress that affects its clinical presentation is that it rarely occurs in isolation. It is now appreciated that complex interactions take place between oxidative and other forms of cell stress, such as endoplasmic reticulum (ER) stress. The clinical manifestation will therefore depend on the balance of metabolic activities in a particular cell type or organ, and so may vary from system to system.

In this review, we will consider the main reactive oxygen species and their generation, the principal antioxidant defences, and then how oxidative stress may be manifested at the maternal–fetal interface during human pregnancy.

## 2 Reactive oxygen species

**Fig. 1:**
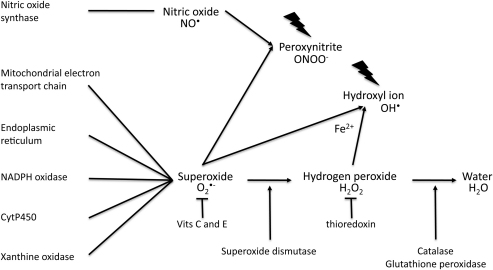
The principal reactive oxygen species, their potential origins and detoxification pathways. NADPH, nicotinamide adenine dinucleotide phosphate.

The term ‘reactive oxygen species’ is applied to both free radicals and their non-radical intermediates. Free radicals are defined as species containing one or more unpaired electrons, and it is this incomplete electron shell that confers their high reactivity. Free radicals can be generated from many elements, but in biological systems it is those involving oxygen and nitrogen that are the most important (Fig. 1).

Under physiological conditions, the most common oxygen free radical is the superoxide anion (O_2_^•−^), and mitochondria are considered the principal source.^3^ The transfer of electrons along the enzymes of the respiratory chain is not totally efficient, and leakage of electrons on to molecular oxygen, in particular from complexes I and III, results in the formation of O_2_^•−^. The rate of formation is determined by the number of electrons present on the chain, and so is elevated under conditions of hyperoxia and of raised glucose, as in diabetes. Paradoxically, it is also increased under conditions of hypoxia, when the reduced availability of oxygen to act as the final electron acceptor for complex IV causes electrons to accumulate. Under normal conditions, 2% of oxygen consumed is converted to O_2_^•−^ in the mitochondria rather than being reduced to water. Because of its charge, O_2_^•−^ is membrane impermeable, and so remains within the mitochondrial matrix.

Similarly, superoxide can also be generated through leakage of electrons from the shorter electron transport chain within the ER.^4^ The formation of disulphide bonds during protein folding is an oxidative process, and about 25% of O_2_•^−^ within cells is generated within the ER. This can increase in cells with a high secretory output, and also under conditions of ER stress when repeated attempts to refold misfolded proteins may take place.

Other sources of superoxide under physiological conditions include the enzymes nicotinamide adenine dinucleotide phosphate (NADPH) oxidase, which generates substantial quantities throughout pregnancy but particularly in early pregnancy,^5^ cytochrome P450, and other oxido-reductases. Hence, various growth factors, drugs and toxins cause increased generation of ROS.^2^ Under pathological conditions, the enzyme xanthine dehydrogenase becomes an important contributor. This enzyme degrades purines, xanthine and hypoxanthine to uric acid and, under normal conditions, uses NAD^+^ as the electron recipient. However, under hypoxic conditions it is proteolytically cleaved to the oxidase form, which donates electrons to molecular oxygen. This enzyme plays a key role in the reperfusion phase of ischaemia–reperfusion injury, when its action is augmented by the build up of hypoxanthine as a result of ATP breakdown during the hypoxic period.

Superoxide is detoxified by the superoxide dismutase enzymes, which convert it to hydrogen peroxide. Hydrogen peroxide is not a free radical, and so is less reactive than O_2_•^−^. However, it comes under the term of ROS as it is intimately involved in the generation and detoxification of free radicals. As it is non-polar, it is able to diffuse through cell and organelle membranes, and hence acts widely as a second messenger in signal transduction pathways. Hydrogen peroxide is in turn detoxified to water by the enzymes catalase and glutathione peroxidase. It is important that the antioxidant enzymes act in concert, as an imbalance in the concentrations of O_2_•^−^ and hydrogen peroxide can result in the formation of the much more dangerous hydroxyl ion (OH•). This reaction is catalysed by free ferrous ions in the Fenton reaction. The hydroxyl ion has an estimated life of 10^−9^ s,^1^ and reacts with any biological molecule in its immediate vicinity in a diffusion-limited manner. Because it is so highly reactive there is no known scavenger of OH•.

Excessive generation of superoxide can also lead to interactions with nitric oxide (NO•) to form peroxynitrite (ONOO^−^). Peroxynitrite is a powerful pro-oxidant. As it is capable of diffusing up to 5 μm, it may affect neighbouring cells.^6^

## 3 Antioxidant defences

Enzymatic and non-enzymatic defences inhibit oxidant attack. The enzymatic defences all have a transition metal at their core, capable of taking on different valences as they transfer electrons during the detoxification process. Two isoforms of superoxide dismutase convert O_2_•^−^ to hydrogen peroxide, the manganese form that is restricted to the mitochondria, and the copper and zinc form that is located in the cytosol. The hydrogen peroxide is then broken down to water by the actions of catalase or glutathione peroxidase, a tetrameric selenoprotein.

The activity of glutathione peroxidase depends on the presence of reduced glutathione (GSH) as a hydrogen donor. Glutathione is the major cellular thiol redox buffer in cells, and is synthesised in the cytosol from l-glutamate, l-cysteine and glycine. GSH participates in a large number of detoxifying reactions forming glutathione disulfide, which is converted back to GSH by the action of glutathione reductase at the expense of NADPH. The latter is generated through the pentose phosphate pathway, of which glucose-6-phosphate dehydrogenase is the first enzyme. This enzyme is subject to common polymorphisms, and decreased activity may compromise GSH concentrations and lead to embryopathy.^7^

The non-enzymatic defences include ascorbate (vitamin C) and α-tocopherol (vitamin E). These again act in concert, with ascorbate being necessary to regenerate reduced α-tocopherol. In addition, thiol compounds, such a thioredoxin, are capable of detoxifying hydrogen peroxide, but in turn require converting back to the reduced form by thioredoxin reductase. Ceruloplasmin and transferrin also play important roles by sequestering free iron ions and so inhibiting the Fenton reaction and production of OH•.

Polymorphisms in the antioxidant enzymes^7,8^ or dietary restriction of micronutrients, such as selenium, can thus play an important role in predisposing to oxidative stress and complications of pregnancy.^9^

## 4 Biological actions of reactive oxygen species

At homeostatic levels, ROS have diverse actions on cell function,^2^ including activation of redox-sensitive transcription factors and activation of protein kinases. These are described below.

### 4.1 Activation of redox-sensitive transcription factors

Activation of redox-sensitive transcription factors, such as AP-1, p53 and NF-κB^10^ regulate the expression of pro-inflammatory and other cytokines, cell differentiation and apoptosis. Under normal conditions NF-κB is held inactive by the binding of its inhibitory sub-unit IκB. However, under conditions of stress, IκB becomes phosphorylated and dissociates from NF-κB, which then translocates to the nucleus and activates expression of pro-inflammatory and other cytokines. Increased phosphorylation of IκB is observed in term placental explants subjected to hypoxia-reoxygenation *in vitro,* which provides a model for malperfusion of the placenta *in vivo*.^11^ Activation of the pathway is associated with increased tissue levels of the proinflammatory enzyme COX-2, interleukin 1ß, increased secretion of TNF-α, and activation of the apoptotic cascade as evinced by cleavage of caspase 3.^12,13^ All these effects can be blocked by the addition of vitamins C and E or sulfasalazine, an inhibitor of NF-κB activation.

### 4.2 Activation of protein kinases

With activation of protein kinases, cells respond to a variety of extracellular signals and stress through a family of mitogen-activated protein kinases (MAPK). Of this family, ROS-induced activation of extracellular regulated kinases (ERK1/2) generally promotes cell survival and proliferation, whereas stimulation of p38MAPK (p38) and stress-activated protein kinase–c-Jun amino terminal kinases (SAPK–JNK) mostly induces apoptosis. p38 and SAPK–JNK are activated by phosphorylation through an upstream kinase, apoptosis-regulating signal kinase 1 (ASK1). Under normal conditions ASK1 is held inactive by binding to thioredoxin, but O_2_•^−^ is capable of oxidising the thiol groups in the latter, leading to a conformational change and its release. Increased phosphorylation of p38, but not SAPK, is observed in the term placenta after labour compared with control participants delivered by caesarean section.^13^ ASK1 is also activated in explants exposed to either hypoxia-reoxygenation or hydrogen peroxide, and is inhibited by addition of vitamins C and E.^13^ Activation is associated with increased levels of the soluble receptor for vascular endothelial growth factor (sFlt-1), which has been implicated in the pathogenesis of pre-eclampsia. Levels of sFlt-1 can be reduced by the addition of vitamins C and E, or inhibitors of the p38 pathway. They can also be reduced by the addition of sulfasalazine, which indicates considerable interactions and mutual reinforcement between the NF-κB and MAPK signalling pathways in the placenta.^13^

The above responses may be considered as physiological adaptive changes to alterations in the environment aimed at restoring homeostasis. More severe attack by ROS may lead to more extensive and irreparable cell damage, resulting ultimately in cell death through necrosis or apoptosis. These more pathological effects are mediated by opening of ion channels, lipid peroxidation, protein modifications and DNA oxidation. These are discussed below.

### 4.3 Opening of ion channels

Imbalances of ROS lead to loss of intracellular Ca^2+^ homeostasis, with release of Ca^2+^ ions from the endoplasmic reticulum and other stores. The calcium concentration within the ER lumen is much higher than in the cytosol, reaching millimolar levels. This concentration is maintained by pumps belonging to the sarco and endoplasmic reticulum calcium ATPase family, and is necessary for the correct functioning of the protein-folding machinery. ROS are able to activate calcium release channels in the ER membrane, which include the inositol-1,4,5,triphosphate receptor (IP_3_R) and the ryanodine receptor.^14^

The resultant release of Ca^2+^ from the ER will activate diverse Ca^2+^-sensitive processes within the cell,^14,15^ including many of the signalling pathways above. It also has a profound effect on function. The loss of chaperone activity results in the accumulation of misfolded proteins within the lumen, leading to further generation of ROS as attempts are made to refold them.^4^ The accumulation will also stimulate the unfolded protein response (UPR), a highly conserved set of signalling pathways that aim to restore homeostasis, but, if this fails, will stimulate apoptosis.^16,17^ The relationship between oxidative and ER stress will be considered in greater detail later.

The rise in cytosolic Ca^2+^ ion concentration will also adversely affect mitochondrial function, including an increase in their own production of ROS and opening of the permeability transition pore. Opening of the membrane permeability transition is promoted synergistically by increased Ca^2+^ ions and oxidation of the thiol groups on proteins in the inner mitochondrial membrane.^18^ As a result, the mitochondrial membrane potential and ATP synthesis collapse. If mitochondria throughout the cell are affected, ATP concentrations fall precipitously, ionic homeostasis is lost and the cell undergoes primary necrosis. Involvement of a more limited number of organelles, or transient opening of the pore, may allow ATP to be maintained at levels sufficient to permit apoptosis to occur instead.^19^

### 4.4 Lipid peroxidation

Hydroxyl radicals are capable of causing lipid peroxidation in the plasma membrane or that of any organelle that contains large quantities of polyunsaturated fatty acid side chains. By abstracting hydrogen from the hydrocarbon side-chain of a fatty acid, they create a carbon-centred radical, C•. If oxygen is present, this may react to form a peroxyl radical (–C–O–O•), which in turn is capable of abstracting hydrogen from an adjacent fatty acid, so propagating the reaction.

Because vitamin E is lipid-soluble and possesses a hydrophobic tail, it tends to accumulate within the interior of lipid membranes. Here, it acts as the most important chain-breaker, as it reacts with lipid peroxyl radicals about four times faster than they can react with adjacent fatty acid side chains.^1^

Evidence of lipid peroxidation can be detected using antibodies directed against one of the principal products, 4-hydroxynonenal. It can be efficiently detoxified in cells by the glutathione S-transferase group of enzymes, but high levels are associated with loss of membrane fluidity and function, and activation of the apoptotic cascade.

### 4.5 Protein modifications

Amino acids, both free and in proteins, are a target for oxidative damage. Direct oxidation of the side chains leads to the formation of carbonyl groups (aldehydes and ketones), and proline, argenine, lysine and threonine are particularly vulnerable to attack.^20^ The carbonyl products are stable, and their detection using enzyme-linked immunosorbent assay or western blotting is the most commonly used method to assay protein oxidation.

Abstraction of hydrogen ions from the thiol group of cysteine can lead to the formation of disulfide bonds and abnormal protein folding, in a manner analagous to the activation of ASK1. Abnormal folding can lead to loss of function, but also protein aggregation and cell death.

Finally, peroxynitrite will react with tyrosine residues to form 3-nitrotyrosine, which can again be detected immunohistochemically. At physiological levels, protein nitration is thought to be a selective and reversible process that leads to activation in a manner analogous to phosphorylation, but at higher levels can be detrimental. Protein nitration in the placenta can therefore have diverse effects, with both gain and loss of function.^21^

### 4.6 DNA oxidation

DNA is attacked principally by OH• radicals, and a variety of products can be generated through reactions with either the DNA bases or the deoxyribose sugars.^1^ For example, OH• can add on to guanine to produce 8-hydroxy-2'-deoxyguanosine, which may be measured biochemically and detected immunohistochemically. Attacks on the sugar moieties may cause strand breakages, whereas those on histone proteins may lead to cross-linkages that interfere with chromatin folding, DNA repair and transcription. Mutation or aberrant gene expression may therefore result.

Mitochondrial DNA is particularly vulnerable to ROS attack owing to its proximity to the site of O_2_•^−^ generation from the electron transport chain, the lack of histone protection, and the minimal repair mechanisms that exist. Consequently, damage to mitochondrial DNA is extensive even under normal conditions, and mutations occur at five to 10 times the rate seen in nuclear DNA.^22^ As mitochondrial DNA encodes several proteins, including enzymes of the electron transport chain, mutations may lead to impaired energy production and the risk of further electron leakage, compounding the original stress.

## 5 Relationship between oxidative stress and endoplasmic reticulum stress

Because ROS can activate so many cell processes, it is not surprising that oxidative stress rarely occurs in isolation, but is usually accompanied by other forms of cell stress. As explained above, close interactions take place between ROS, mitochondrial and ER function, mediated through Ca^2+^ release that potentially constitute a feed–forward system.^23^

The ER has recently become recognised as a major centre for the co-ordination of cellular responses to a variety of stressors. In part, this is because protein synthesis accounts for about 30% of the energy expenditure of a cell, and so needs to be finely tuned to oxygen and nutrient availability. Under conditions of stress, the UPR aims to restore homeostasis by a co-ordinated set of responses that reduce the burden of misfolded proteins. Firstly, it blocks the influx of new proteins through phosphorylation and inhibition of the eukaryotic initiation factor eIF2α, which regulates the initiation of translation. Secondly, it increases the expression of the ER chaperone proteins GRP78 and 94 in an attempt to sequester or refold the misfolded proteins. Thirdly, it increases the synthesis of ER cisternae, and finally it stimulates the ER-associated degradation machinery.^16,17^ If these attempts fail and ER stress persists, then the UPR will activate the apoptotic cascade through increased expression of the C/EBP homologous protein in order to eliminate the cell.

A further aspect of ER stress that is of particular relevance to pregnancy is its relationship to pro-inflammatory pathways. Activation of these pathways can occur through at least two mechanisms. The first involves the NF-κB pathway. One of the three signalling transducers activated during initiation of the UPR, the inositol-requiring protein-1 (Ire1) pathway, has dual actions. Ire1 contains an endoribonuclease domain, which when activated splices XBP-1 pre-mRNA to produce the transcription factor, XBP-1, that stimulates transcription of genes regulating the breakdown of misfolded proteins and ER biogenesis. Ire1 also contains a Ser/Thr kinase domain that is capable of activating the NF-κB pathway through phosphorylation of IκB, and the p38 and SAPK–JNK pathways through ASK1. In addition, activation of NF-κB can also occur as a consequence of inhibition of protein translation secondary to phosphorylation of eIF2α, as the half-life of IκB is shorter than that of NF-κB.^24^

The second mechanism is not related directly to activation of the UPR, but involves proteins whose predicted structure is similar to that of one of the other signal transducing proteins, activating transcription factor.^17^ In the liver, cyclic AMP response element binding protein hepatocyte is thought to reside in the ER membrane but is released upon ER stress to activate acute-phase genes. This leads to the increased secretion of acute-phase response proteins, such as C-reactive protein.

Of the two stresses, oxidative and ER stress, it is likely that latter will be detected at lower levels of insults as the UPR is a homeostatic mechanism, whereas the commonly used markers of oxidative stress reflect cell injury.

## 6 Oxidative stress at the maternal–fetal interface

**Fig. 2:**
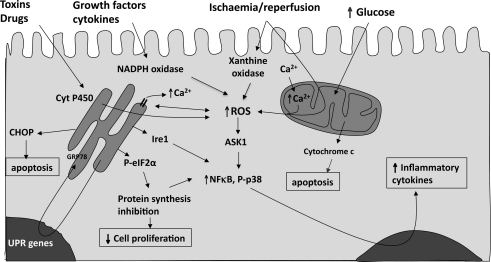
How reactive oxygen species may be generated within the syncytiotrophoblast, and the principal consequences for the function of the tissue. CHOP, C/EBP homologous protein; NADPH, nicotinamide adenine dinucleotide phosphate; ROS, reactive oxygen species; UPR, unfolded protein response.

From the preceding descriptions, it can be seen that oxidative stress may induce a range of cellular responses depending upon the severity of the insult and the compartment in which the ROS are generated. Some of the more major signalling pathways involved and potential outcomes are presented in Fig. 2. The close interaction between oxidative stress and ER stress is important when considering potential therapeutic interventions, as there will be little benefit in addressing one of the stresses without addressing the other.

Oxidative stress is manifested at the maternal–fetal interface from early pregnancy onwards. It plays a role in both the normal development of the placenta as well as in the pathophysiology of complications such as miscarriage, pre-eclampsia, intrauterine growth restriction (IUGR), and premature rupture of the membranes.^25,26^ The more important aspects will be considered in turn.

### 6.1 Oxidative stress and placental remodelling

The human placenta is unique because villi form initially over the entire surface of the chorionic sac. Starting towards the end of the first trimester, however, the villi over the superficial pole regress to leave the definitive discoid placenta. It is now recognised that oxidative stress plays a central role in this process, and as regression occurs in all pregnancies this can be considered physiological.

Our appreciation of the intrauterine environment during human early pregnancy has undergone a radical revision over the last two decades. It is now accepted that placental development occurs in a relatively low oxygen concentration, supported by secretions from the endometrial glands rather than the maternal circulation ^27,28^ We have speculated that this environment protects the developing embryo from oxygen free radical mediated teratogenesis.^29^ Maternal arterial blood is prevented from entering the intervillous space of the placenta by plugs of extravillous cytotrophoblast cells that invade down the mouths of the uterine spiral arteries as part of the process of physiological conversion.^30,31^ The maternal intraplacental circulation is only fully established towards the end of the first trimester, when these plugs dislocate through a mechanism that is currently unknown. Ultrasonographic evidence has shown that the circulation starts preferentially in the periphery of the placenta, where trophoblast invasion is the least extensive, and progressively extends into the central region.^32^

Onset of the circulation is associated with a three-fold rise in the oxygen concentration within the placenta.^27^ This will stimulate higher rates of generation of ROS, particularly in the critical syncytiotrophoblastic layer, which contains low concentrations of the principal antioxidant enzymatic defences, copper and zinc superoxide dismutase and catalase.^33,34^ Consequently, villi sampled from the peripheral region of the placenta demonstrate elevated levels of the chaperone protein HSP70, nitrotyrosine residues indicative of peroxynitrite formation, and morphological evidence of degenerative changes within the syncytiotrophoblast compared with those from the central region.^32^ Molecular evidence confirms that the apoptotic cascade is activated in the peripheral villi and, over an extended period, this would be sufficient to account for their regression.

### 6.2 Oxidative stress and spontaneous miscarriage

In cases of miscarriage, onset of the maternal intraplacental circulation is both precocious and disorganised compared with normal ongoing pregnancies.^27,32^ Thus it starts at an earlier stage, and occurs randomly throughout the placenta. This is probably because, in 70% of these cases, extravillous trophoblast invasion is superficial, and consequently plugging of the spiral arteries is less complete.^35,36^ As might be expected, levels of HSP70 and nitrotyrosine are increased in villi sampled from the central region of these placentas as well as in the periphery.^32,37^ The apoptotic index is also increased compared with control placentas of a similar gestational age, and there is morphological evidence of degenerate syncytiotrophoblast sloughing off in some areas. In these cases, it seems that overwhelming oxidative stress causes widespread destruction of the trophoblast, incompatible with an ongoing pregnancy.

In confirmation of these findings, lipid peroxides have been shown to be increased in villous and decidual tissues, and the serum of women undergoing pregnancy loss.^7,38–40^ Also, elevated concentrations of protein carbonyls and DNA damage have been reported in cases of complete hydatidiform mole, where plugging of the spiral arteries by extravillous trophoblast is equally deficient.^41^

Onset of the maternal circulation clearly represents an oxidative challenge to the human placenta. Even in normal pregnancies, a burst of oxidative stress in the placenta takes place at 10–12 weeks of gestation, which resolves as the placental tissues adapt to their new oxygen environment. That adaptation involves an increase in the expression and activity of the principal antioxidant enzymes.^27^ The efficacy of the antioxidant defences provides the other half of the pro-oxidant-antioxidant balance that determines the oxidative status of a tissue. Thus, polymorphisms in the enzymes detoxifying ROS have been linked to an increased risk of miscarriage.^8,42,43^ Equally, some evidence shows that selenium deficiency, which will reduce the efficacy of glutathione peroxidase, is associated with miscarriage.^9,44^ Conversely, it has been reported that administration of the antioxidant *N*-acetyl cysteine to women suffering recurrent pregnancy loss improves the take-home baby rate.^45^

Further research is therefore required to test prospectively whether dietary supplementation with micronutrients or antioxidants can reduce the rate of spontaneous miscarriage.

### 6.3 Placental oxidative stress in pre-eclampsia

Normal pregnancy is said to be a condition of oxidative stress, as circulating levels of oxidised low-density lipoproteins increase and the total antioxidant capacity in pregnant women decreases compared with non-pregnant women.^46^ Pregnancy is also associated with a systemic inflammatory response, as evinced by activation of peripheral granulocytes, monocytes and lymphocytes during the third trimester, all of which produce ROS.^47^ These states are therefore obviously interlinked, and capable of forming dangerous feed–forward systems.

Oxidative stress and the systemic inflammatory response are observed to a much greater degree in pre-eclampsia.^48^ There is irrefutable evidence of placental oxidative stress in cases of early onset pre-eclampsia, including increased concentrations of protein carbonyls, lipid peroxides, nitrotryosine residues and DNA oxidation.^49,50^ The cause for the oxidative stress is thought to be vascular, because early onset pre-eclampsia is associated with deficient conversion of the spiral arteries. In particular, the myometrial segments of the arteries are adversely affected.^51–53^ As the myometrial segment contains a highly contractile portion of the artery, we have proposed that failure to convert this section results in intermittent perfusion of the placenta, and a low-grade ischaemia-reperfusion type injury.^11,25^ In support of this hypothesis, we have shown that hypoxia-reoxygenation *in vitro* is a potent inducer of oxidative stress in term placental explants, much more than hypoxia alone.^11^ Exposure of explants to changes in oxygenation causes generation of ROS within the trophoblast and endothelial cells, as shown by fluorescent markers and the formation of nitrotyrosine residues in a pattern matching closely to that seen in pre-eclamptic placentas. Furthermore, labour, in which the placenta is exposed to repeated episodes of ischaemia–reperfusion, induces high levels of oxidative stress.^54^ This is associated with increased xanthine oxidase activity,^55^ and changes in gene expression that mimic those seen in pre-eclampsia.^54^

Another potential source of oxidative stress in pre-eclampsia is autoantibodies against the angiotensin AT1 receptor.^56^ These antibodies stimulate NADPH oxidase, leading to an increase in ROS production.

In the classic two-stage model of pre-eclampsia, oxidative stress induced in the placenta is thought to cause the release of factors into the maternal circulation that stimulate the inflammatory response and activation of the maternal endothelial cells.^48^ Many placental factors have been implicated, including microparticulate apoptotic debris, pro-inflammatory cytokines and angiogenic factors.^13,48,57^ To date, no single factor has been indentified that can account for all cases of pre-eclampsia. This may indicate that the true causation has yet to be discovered, or that the syndrome is capable of being initiated by a variety of different stimuli.

Early onset pre-eclampsia is almost invariably associated with IUGR, and we have recently reported morphological and molecular evidence of high levels of ER stress in these placentas.^58^ Strong phosphorylation of eIF2α will cause suppression of protein synthesis. Consequently, the level of cyclin D1, a kinase that plays a central role in the regulation of cell proliferation, is significantly reduced. Induction of similar stress in trophoblast-like cell lines causes a reduction in their proliferation rate. Increased expression of the pro-apoptotic C/EBP homologous protein also takes place. This combination would provide a sufficient cause for the placental growth restriction observed. In addition, the high levels of ER stress may contribute to the inflammatory response by stimulating the p38 and NF-κB pathways. Hence, we speculate that both ER stress and oxidative stress contribute to the placental pathophysiology in pre-eclampsia in a mutually reinforcing fashion.^50^

This weight of evidence provides a strong rationale for considering antioxidants as a potential therapy for pre-eclampsia. Unfortunately, recent trials of vitamins C and E in a number of different settings have not proved successful.^59–61^ This failure contrasts strikingly to the beneficial effects observed on specific signal transduction pathways and placental outcomes to oxidative challenge *in vitro*.^12^ The difference may result from the ability of the vitamins to access the relevant trophoblast cell compartment in the necessary concentration *in vivo*. Alternatively, in the clinical trials, the antioxidants are only given once pregnancy is established, by which time the feed–forward cycles may already be established. It is notable that multivitamin usage during the periconceptional period is associated with a reduced risk of pre-eclampsia among lean or normal weight women.^62,63^ Conversely, women with a low dietary intake of vitamin C have been reported to have a trend towards increased risk.^64^ These data suggest that more attention needs to be focused on ensuring optimal health and diet in women planning to conceive.

### 6.4 Placental oxidative stress in intra-uterine growth restriction

IUGR can have many causes, but most cases that have no genetic or infectious cause are thought to arise from compromise of the maternal circulation to the placenta. This conclusion is based on the common association with high resistance uterine arterial waveforms, but also on earlier morphological studies of the spiral arteries. The investigators reported deficient physiological conversion of the arteries as in pre-eclampsia, but to a lesser degree, especially in the myometrial segment.^52^ Indeed, a positive correlation was observed in one study between the birth weight and the degree of conversion.^51^ One might predict, therefore, that the vascular insult is less severe in cases of IUGR alone, but this requires confirmation.

In contrast to pre-eclampsia, surprisingly little attention has been paid to the role of placental oxidative stress in cases of IUGR alone. The scanty evidence available indicates that the level of stress is either similar to the normal control or intermediate between a normal control and an early onset pre-eclamptic placenta,^50,65^ These findings are in keeping with the predicted vascular insult above. However, we do still observe morphological and molecular evidence of ER stress in these placentas.^58^ The level of stress is lower than in pre-eclampsia, with no evidence of activation of the apoptotic cascade. But it is sufficient to cause phosphorylation of eIF2α and suppression of protein synthesis, with a reduction in the level of cyclin D1. ER stress can be induced by malperfusion through disturbances of Ca^2+^ homeostasis. Hence, we speculate that chronic low-level ER stress may occur from the time of onset of the maternal circulation onwards, and leads to a lower growth trajectory of the placenta. This is consistent with the reduced rate of growth of the placenta observed with serial ultrasound scans in these cases^66^

### 6.5 Preterm premature rupture of the membranes

Oxidative stress has been implicated in preterm premature rupture of the membranes, a condition associated with proteolytic degradation of the collagen fibres in the chorio-amnion. In this condition, increase in the generation of ROS may arise from infection and inflammation, cigarette smoking, vaginal bleeding and the release of free iron, and cocaine abuse which leads to ischaemia–reperfusion.^67^ It has also been shown that deficient conversion of the spiral arteries predisposes to this condition.^68^ Exposure of the chorio-amnion to O_2_•^−^ *in vitro* results in up-regulation of matrix metalloproteinase-9, which can be suppressed by the antioxidant *N*-acetylcysteine.^67^ Intriguingly, pre-treatment with vitamins C and E protects the membranes against oxidative attack *in vitro*, but whether they can be effective *in vivo* remains to be determined.

## 7 Conclusion

The role of ROS in human placentation and complications of pregnancy is a relatively recent field, but one that is expanding rapidly. It is increasingly apparent that they play a central role in many signal transduction pathways, and it is important to recognise that homeostatic concentrations are present in all tissues. Excessive antioxidant intake could potentially threaten and inactivate these vital signalling responses, with adverse consequences. At excessive concentrations, whether caused by increased generation or reduced detoxification, ROS can cause widespread and indiscriminate damage to cells and tissues. ROS can be generated through many pathways within cells, but the mitochondria, ER and enzymes such as NADPH oxidase are the most important sources. These pathways can respond to a variety of stimuli, but arguably the most important ones for pregnancy are perturbations in the maternal blood supply to the placenta and inflammation. Because of interactions between downstream signalling pathways, oxidative stress rarely occurs in isolation, but usually interacts with ER and other forms of cell stress. We propose that spontaneous miscarriage, early onset pre-eclampsia and IUGR represent a spectrum of placental oxidative stress-related disorders, secondary to deficient trophoblast invasion and physiological conversion of the spiral arteries. Although trials of antioxidant interventions have not proved successful in pre-eclampsia, the rationale for this treatment remains strong, and further research of the benefits of periconceptional supplementation is required.

**Practice points**

• The maternal circulation to the placenta is not fully established until towards the end of the first trimester in normal human pregnancies.
• The establishment of the maternal circulation is a progressive phenomenon that modulates the development of the definitive placenta.
• That deficient conversion of the maternal spiral arteries underlies placental-related complications of pregnancy, such as miscarriage and pre-eclampsia.
• Fluctuations in maternal blood flow to the placenta may be of greater pathological consequence than hypoxia alone.
• The pathophysiology of miscarriage is linked to a premature and overwhelming entry of maternal blood inside the placenta, whereas that of pre-eclampsia is linked to an ischaemia-reperfusion phenomenon.

**Research agenda**

To establish the following:

• Factors that regulate spiral arterial remodelling and unplugging of the spiral arteries
• Importance of polymorphisms in the antioxidant pathways for complications of pregnancy
• Factors released from the placenta that lead to the maternal inflammatory response
• Importance of preconceptional nutrition to the risk of miscarriage and pre-eclampsia
• Why antioxidants vitamins taken during pregnancy have no effect on rates of pre-eclampsia or miscarriage
• The role of environmental oxidative stress on placental-related disorders of pregnancy.

## References

[1] Halliwell B., Gutteridge J.M.C. (1999). Free radicals in biology and medicine.

[2] Droge W. (2002). Free radicals in the physiological control of cell function. Physiol Rev.

[3] Cadenas E., Davies K.J.A. (2000). Mitochondrial free radical generation, oxidative stress, and aging. Free Rad Biol Med.

[4] Tu B.P., Weissman J.S. (2004). Oxidative protein folding in eukaryotes: mechanisms and consequences. J Cell Biol.

[5] Raijmakers M.T., Burton G.J., Jauniaux E. (2006). Placental NAD(P)H oxidase mediated superoxide generation in early pregnancy. Placenta.

[6] Pacher P., Beckman J.S., Liaudet L. (2007). Nitric oxide and peroxynitrite in health and disease. Physiol Rev.

[7] Nicol C.J., Zielenski J., Tsui L.-C. (2000). An embryoprotective role for glucose-6-phosphate dehydrogenase in developmental oxidative stress and chemical teratogenesis. FASEB J.

[8] Tempfer C., Unfried G., Zeillinger R. (2001). Endothelial nitric oxide synthase gene polymorphism in women with idiopathic recurrent miscarriage. Hum Reprod.

[9] Al-Kunani A.S., Knight R., Haswell S.J. (2001). The selenium status of women with a history of recurrent miscarriage. BJOG.

[10] Arrigo A.P., Kretz-Remy C., Aruoma O.I., Halliwell B. (1998). Regulation of mammalian gene expression by free radicals. Molecular biology of free radicals in human diseases.

[11] Hung T.H., Skepper J.N., Burton G.J. (2001). In vitro ischemia-reperfusion injury in term human placenta as a model for oxidative stress in pathological pregnancies. Am J Pathol.

[12] Cindrova-Davies T., Spasic-Boskovic O., Jauniaux E. (2007). Nuclear factor-kappa B, p38, and stress-activated protein kinase mitogen-activated protein kinase signaling pathways regulate proinflammatory cytokines and apoptosis in human placental explants in response to oxidative stress: effects of antioxidant vitamins. Am J Pathol.

[13] Cindrova-Davies T. (2009). From placental oxidative stress to maternal endothelial dysfunction. Placenta Suppl A.

[14] Hool L.C., Corry B. (2007). Redox control of calcium channels: from mechanisms to therapeutic opportunities. Antioxid Redox Signal.

[15] Berridge M.J., Bootman M.D., Roderick H.L. (2003). Calcium signalling: dynamics, homeostasis and remodelling. Nat Rev Mol Cell Biol.

[16] Xu C., Bailly-Maitre B., Reed J.C. (2005). Endoplasmic reticulum stress: cell life and death decisions. J Clin Invest.

[17] Ron D., Walter P. (2007). Signal integration in the endoplasmic reticulum unfolded protein response. Nat Rev Mol Cell Biol.

[18] Kowaltowski A.J., Castilho R.F., Vercesi A.E. (2001). Mitochondrial permeability transition and oxidative stress. FEBS Lett.

[19] Leist M., Single B., Castoldi A.F. (1997). Intracellular adenosine triphosphate (ATP) concentration: a switch in the decision between apoptosis and necrosis. J Exp Med.

[20] Dalle-Donne I., Rossi R., Giustarini D. (2003). Protein carbonyl groups as biomarkers of oxidative stress. Clin Chim Acta.

[21] Myatt L. (2010). Review: reactive oxygen and nitrogen species and functional adaptation of the placenta. Placenta.

[22] Richter C., Park J.-W., Ames B.N. (1988). Normal oxidative damage to mitochondrial and nuclear DNA is extensive. Proc Nat Acad Sci USA.

[23] Zhang K. (2010). Integration of ER stress, oxidative stress and the inflammatory response in health and disease. Int J Clin Exp Med.

[24] Zhang K., Kaufman R.J. (2008). From endoplasmic-reticulum stress to the inflammatory response. Nature.

[25] Burton G.J., Jauniaux E. (2004). Placental oxidative stress; from miscarriage to preeclampsia. J Soc Gynecol Invest.

[26] Jauniaux E., Poston L., Burton G.J. (2006). Placental-related diseases of pregnancy: involvement of oxidative stress and implications in human evolution. Hum Reprod Update.

[27] Jauniaux E., Watson A.L., Hempstock J. (2000). Onset of maternal arterial bloodflow and placental oxidative stress; a possible factor in human early pregnancy failure. Am J Pathol.

[28] Burton G.J., Watson A.L., Hempstock J. (2002). Uterine glands provide histiotrophic nutrition for the human fetus during the first trimester of pregnancy. J Clin Endocrinol Metab.

[29] Burton G.J., Hempstock J., Jauniaux E. (2003). Oxygen, early embryonic metabolism and free radical-mediated embryopathies. Reprod BioMed Online.

[30] Hustin J., Schaaps J.P. (1987). Echographic and anatomic studies of the maternotrophoblastic border during the first trimester of pregnancy. Am J Obstet Gynecol.

[31] Burton G.J., Jauniaux E., Watson A.L. (1999). Maternal arterial connections to the placental intervillous space during the first trimester of human pregnancy; the Boyd Collection revisited. Am J Obstet Gynecol.

[32] Jauniaux E., Hempstock J., Greenwold N. (2003). Trophoblastic oxidative stress in relation to temporal and regional differences in maternal placental blood flow in normal and abnormal early pregnancies. Am J Pathol.

[33] Watson A.L., Palmer M.E., Jauniaux E. (1997). Variations in expression of copper/zinc superoxide dismutase in villous trophoblast of the human placenta with gestational age. Placenta.

[34] Watson A.L., Skepper J.N., Jauniaux E. (1998). Changes in the concentration, localisation and activity of catalase within the human placenta during early gestation. Placenta.

[35] Hustin J., Jauniaux E., Schaaps J.P. (1990). Histological study of the materno-embryonic interface in spontaneous abortion. Placenta.

[36] Jauniaux E., Zaidi J., Jurkovic D. (1994). Comparison of colour doppler features and pathologic findings in complicated early pregnancy. Hum Reprod.

[37] Hempstock J., Jauniaux E., Greenwold N. (2003). The contribution of placental oxidative stress to early pregnancy failure. Hum Pathol.

[38] Sugino N., Kakata M., Kashida S. (2000). Decreased superoxide dismutase expression and increased concentrations of lipid peroxide and prostaglandin F(2alpha) in the decidua of failed pregnancy. Mol Hum Reprod.

[39] Biri A., Kavutcu M., Bozkurt N. (2006). Investigation of free radical scavenging enzyme activities and lipid peroxidation in human placental tissues with miscarriage. J Soc Gynecol Investig.

[40] Toy H., Camuzcuoglu H., Camuzcuoglu A. (2010). Decreased serum prolidase activity and increased oxidative stress in early pregnancy loss. Gynecol Obstet Invest.

[41] Harma M. (2006). Defective placentation and resultant oxidative stress play a similar role in complete hydatidiform mole to that in preeclampsia and early pregnancy loss. Med Hypotheses.

[42] Sata F., Yamada H., Kondo T. (2003). Glutathione S-transferase M1 and T1 polymorphisms and the risk of recurrent pregnancy loss. Mol Hum Reprod.

[43] Sata F., Yamada H., Yamada A. (2003). A polymorphism in the CYP17 gene relates to the risk of recurrent pregnancy loss. Mol Hum Reprod.

[44] Zachara B.A., Dobrzynski W., Trafikowska U. (2001). Blood selenium and glutathione peroxidases in miscarriage. BJOG.

[45] Amin A.F., Shaaban O.M., Bediawy M.A. (2008). N-acetyl cysteine for treatment of recurrent unexplained pregnancy loss. Reprod Biomed Online.

[46] Belo L., Caslake M., Santos-Silva A. (2004). LDL size, total antioxidant status and oxidised LDL in normal human pregnancy: a longitudinal study. Atherosclerosis.

[47] Redman C.W., Sargent I.L. (2003). Pre-eclampsia, the placenta and the maternal systemic inflammatory response–a review. Placenta.

[48] Redman C.W., Sargent I.L. (2009). Placental stress and pre-eclampsia: a revised view. Placenta.

[49] Myatt L., Cui X. (2004). Oxidative stress in the placenta. Histochem Cell Biol.

[50] Burton G.J., Yung H.W., Cindrova-Davies T. (2009). Placental endoplasmic reticulum stress and oxidative stress in the pathophysiology of unexplained intrauterine growth restriction and early onset preeclampsia. Placenta.

[51] Gerretsen G., Huisjes H.J., Elema J.D. (1981). Morphological changes of the spiral arteries in the placental bed in relation to pre-eclampsia and fetal growth retardation. Br J Obstet Gynaecol.

[52] Khong T.Y., De Wolf F., Robertson W.B. (1986). Inadequate maternal vascular response to placentation in pregnancies complicated by pre-eclampsia and by small-for-gestational age infants. Br J Obstet Gynaecol.

[53] Meekins J.W., Pijnenborg R., Hanssens M. (1994). A study of placental bed spiral arteries and trophoblast invasion in normal and severe pre-eclamptic pregnancies. Br J Obstet Gynaecol.

[54] Cindrova-Davies T., Yung H.W., Johns J. (2007). Oxidative stress, gene expression, and protein changes induced in the human placenta during labor. Am J Pathol.

[55] Many A., Roberts J.M. (1997). Increased xanthine oxidase during labour-implications for oxidative stress. Placenta.

[56] Dechend R., Viedt C., Muller D.N. (2003). AT1 receptor agonistic antibodies from preeclamptic patients stimulate NADPH oxidase. Circulation.

[57] Redman C.W., Sargent I.L. (2005). Latest advances in understanding preeclampsia. Science.

[58] Yung H.W., Calabrese S., Hynx D. (2008). Evidence of placental translation inhibition and endoplasmic reticulum stress in the etiology of human intrauterine growth restriction. Am J Pathol.

[59] Poston L., Briley A.L., Seed P.T. (2006). Vitamin C and vitamin E in pregnant women at risk for pre-eclampsia (VIP trial): randomised placebo-controlled trial. Lancet.

[60] Roberts J.M., Myatt L., Spong C.Y. (2010). Vitamins C and E to prevent complications of pregnancy-associated hypertension. N Engl J Med.

[61] Xu H., Perez-Cuevas R., Xiong X. (2010). An international trial of antioxidants in the prevention of preeclampsia (INTAPP). Am J Obstet Gynecol.

[62] Bodnar L.M., Tang G., Ness R.B. (2006). Periconceptional multivitamin use reduces the risk of preeclampsia. Am J Epidemiol.

[63] Catov J.M., Nohr E.A., Bodnar L.M. (2009). Association of periconceptional multivitamin use with reduced risk of preeclampsia among normal-weight women in the Danish National Birth Cohort. Am J Epidemiol.

[64] Klemmensen A., Tabor A., Osterdal M.L. (2009). Intake of vitamin C and E in pregnancy and risk of pre-eclampsia: prospective study among 57 346 women. BJOG.

[65] Wikstrom A.K., Nash P., Eriksson U.J. (2009). Evidence of increased oxidative stress and a change in the plasminogen activator inhibitor (PAI)-1 to PAI-2 ratio in early-onset but not late-onset preeclampsia. Am J Obstet Gynecol.

[66] Hafner E., Metzenbauer M., Hofinger D. (2003). Placental growth from the first to the second trimester of pregnancy in SGA-foetuses and pre-eclamptic pregnancies compared to normal foetuses. Placenta.

[67] Woods J.R. (2000). Reactive oxygen species and preterm premature rupture of membranes-a review. Placenta.

[68] Kim Y.M., Chaiworapongsa T., Gomez R. (2002). Failure of the physiologic transformation of the spiral arteries in the placental bed in preterm premature rupture of membranes. Am J Obstet Gynecol.

